# Boosting Food System Stability Through Technological Progress in Price and Supply Dynamics

**DOI:** 10.3390/foods14223910

**Published:** 2025-11-15

**Authors:** Nicoleta Mihaela Doran

**Affiliations:** Department of Finance, Banking and Economic Analysis, Faculty of Economics and Business Administration, University of Craiova, 200585 Craiova, Romania; nicoleta.doran@edu.ucv.ro

**Keywords:** technological progress, food price dynamics, supply variability, European Union

## Abstract

This study examines the impact of technological progress on food price dynamics and supply stability across the 27 European Union Member States during 2011–2024. Using a balanced panel dataset, the analysis explores four dependent indicators—consumer food prices, food price inflation, price volatility, and food supply variability—while controlling for trade openness, GDP per capita growth, and population. Technological progress is estimated through panel least squares regression with fixed effects. The results reveal that technological advancement significantly reduces food prices and inflation, suggesting that innovation-driven productivity and efficiency gains stabilize consumer markets. However, its influence on food price volatility and supply variability is statistically insignificant, indicating that innovation alone cannot fully mitigate systemic risks in the European food system. The results provide policy-relevant evidence supporting the integration of technological innovation into food system governance across the European Union. They underline the need for targeted investment and regulatory coordination to translate innovation gains into tangible resilience outcomes, thus offering practical guidance for policymakers and stakeholders involved in implementing the European Green Deal and the Farm to Fork Strategy.

## 1. Introduction

The stability of food systems has become a strategic priority for the European Union (EU), reflecting the combined pressures of climate change, energy crises, geopolitical disruptions, and post-pandemic market volatility. Recent years have seen substantial increases in food prices and disruptions in supply chains, which have amplified inflationary pressures and raised concerns about food security and affordability across Member States. According to the Food and Agriculture Organization (FAO) [1], food price volatility and supply variability are key determinants of nutritional security and economic resilience, particularly during global shocks such as the 2022 energy and fertilizer crises. Ensuring stable and sustainable food systems has therefore become an essential component of the EU’s Green Deal and Farm to Fork Strategy, both of which emphasize technological modernization and digitalization of the agri-food sector [2,3].

In parallel, the European food system is increasingly integrated into global commodity markets, making it more exposed to international price fluctuations and energy dependencies [4]. The interaction between trade openness, supply chain efficiency, and technological capacity has become a crucial factor in determining how national food systems absorb and transmit shocks. Although productivity gains have been achieved in agriculture and manufacturing, disparities persist among EU Member States in terms of technological capacity, market efficiency, and adaptive resilience [5,6]. Understanding how technological progress affects food price inflation, volatility, and supply stability is therefore critical to designing coherent EU policies that enhance both competitiveness and food security.

Technological advancement plays a central role in stabilizing food systems by increasing efficiency, improving supply chain transparency, and reducing losses across production, storage, and distribution stages. Digital tools, precision agriculture, cold-chain infrastructure, and real-time market information can significantly enhance food system responsiveness to shocks [7,8]. Empirical evidence suggests that economies with stronger technological capacities are better equipped to manage fluctuations in energy costs and global demand, maintaining more stable food prices and food availability over time [9]. Consequently, evaluating the link between technological progress and food market stability within the EU offers valuable insights into the region’s long-term resilience and policy effectiveness.

Despite growing attention to the economic and environmental dimensions of food security, the systemic role of technological progress in food system stability remains underexplored in the literature. Previous research has largely focused on agricultural productivity [10], trade integration [11], or policy mechanisms aimed at mitigating price volatility [12]. However, there is limited empirical evidence on how technological advancement—measured through innovation capacity, R&D intensity, and digital infrastructure—affects both price and supply dynamics within integrated European food markets. Furthermore, cross-country analyses quantifying this relationship using harmonized indicators remain scarce, leaving an important empirical gap in the understanding of EU food system resilience. Previous studies have primarily examined innovation impacts in isolation—focusing on agricultural productivity, trade integration, or eco-innovation—without jointly analyzing their influence on both price stability and supply resilience at the EU level. This study addresses that gap by integrating price- and supply-related indicators within a unified empirical framework, covering all 27 Member States from 2011 to 2024. By doing so, it contributes to the existing literature by providing macro-level quantitative evidence on how technological progress stabilizes food prices and interacts with structural factors such as trade openness, income growth, and population. This approach offers a comprehensive and policy-oriented perspective, aligning technological advancement with the EU’s long-term objectives of sustainable and resilient food systems.

The present study aims to examine the relationship between technological progress and food system stability in the 27 EU Member States over the period 2011–2024. Technological progress (TECHP) is measured through the Global Innovation Index (GII), a composite proxy capturing countries’ technological capabilities, research intensity, and innovation performance. Food system stability is evaluated using four key indicators: consumer prices for food (CPFI), food price inflation (FPI), food price volatility (FPV), and food supply variability (FSV). To account for macroeconomic and structural heterogeneity, the models include trade openness (TO), GDP per capita growth (GDP), and population (POP) as control variables. The empirical analysis employs a panel fixed-effects approach with robust standard errors, ensuring comparability across Member States and over time.

The originality of this research lies in its integrated analytical framework, which links technological progress to both price and supply dimensions of food system stability within the European Union. Unlike prior studies that address either productivity or price dynamics in isolation, this paper conceptualizes stability as a multifaceted outcome shaped by technological, economic, and institutional factors. The findings are expected to contribute new quantitative evidence on the stabilizing effects of technological progress in the EU’s agri-food sector, offering policy-relevant insights for enhancing food market resilience and achieving the long-term objectives of the European Green Deal and Farm to Fork Strategy.

## 2. Literature Review

Technological progress and innovation have become central to achieving sustainability, competitiveness, and food security in modern agri-food systems. Recent literature converges toward an integrated understanding of innovation as a multidimensional process that connects technological, institutional, and social transformations. The evolution of the European food sector, under the Green Deal and Farm to Fork Strategy, illustrates how innovation-driven modernization aims to stabilize markets, enhance efficiency, and strengthen resilience against systemic shocks.

Early studies identified a transition from isolated technological improvements toward open and collaborative innovation ecosystems. Rodríguez et al. [13] and Neuberger et al. [14] show that participatory networks and cross-border cooperation enhance knowledge diffusion but remain constrained by national policies. Similarly, Indrawati et al. [15] highlight the dominant role of organizational commitment in fostering green innovation among SMEs. Collectively, these studies emphasize that institutional frameworks and governance structures are as important as technological capacity for innovation success.

From a broader perspective, Dzhunushalieva and Teuber [16] and Chouaibi et al. [17] demonstrate that innovation in the food sector is increasingly tied to corporate governance, ownership structures, and social responsibility. Fortuny-Sicart et al. [18] further conceptualize innovation as a democratic process that can promote inclusiveness and equitable outcomes. These insights position technological progress as part of a socio-political transformation, where participation and accountability shape innovation outcomes.

Digitalization has accelerated this transformation. Wang et al. [19] and Jin et al. [20] show that adopting digital technologies and genetically modified crops enhances productivity and export competitiveness, while Marozzo et al. [21] demonstrate that collaboration between large firms and startups accelerates innovation diffusion. In Europe, Stranieri et al. [22] and Chaparro-Banegas et al. [23] reveal non-linear effects of innovation: lagging regions benefit more from technological upgrading, while advanced ones experience diminishing returns. These findings suggest that technological progress reduces disparities but only under supportive institutional conditions. Escalante and García [24] add that educational innovation remains a vital channel for building innovation capacity.

A growing body of research connects innovation to sustainability. Bor et al. [25] and Wang et al. [26] provide evidence that technological innovation enhances the resilience of food industries, though with decreasing marginal effects. Onumah et al. [27] and Bujor and Avasilcai [28] expand this view by linking open innovation and gender inclusion to social sustainability, confirming that innovation outcomes are context-dependent. Similarly, Reinhardt and Monaco [29] and de Lima et al. [30] stress the role of regulation and patents in steering innovation trajectories, while Onegina et al. [31] highlight persistent disparities among small firms in technological adoption.

In emerging economies, digitalization and education amplify innovation’s positive effects. Thann et al. [32] and David et al. [33] identify technological innovation as a key enabler of sustainability within the water–energy–food nexus. Onumah et al. [34] confirm that gender and credit access determine innovation uptake in Ghana’s fisheries sector. Meanwhile, Joshi et al. [35] and Simon and Young [36] illustrate how post-pandemic recovery and ethical constraints shape innovation pathways in global food chains. Within Europe, Stranieri et al. [37] and Borgia et al. [38] find that geographical indications and targeted policy instruments strengthen competitiveness by promoting sustainable technologies.

Institutional and ecosystem dynamics play a critical mediating role. Bulah et al. [39] and Coletto et al. [40] argue that norms, values, and knowledge spillovers within innovation ecosystems are decisive for technological diffusion. Wei et al. [41] and Xu et al. [42] provide further evidence that environmental regulations indirectly enhance food security through the mediating role of innovation. These studies collectively reinforce the governance dimension of technological progress—innovation succeeds when supported by coherent policies and institutional trust.

At the environmental frontier, climate-smart and digital innovations are reshaping agri-food production. Konfo et al. [43] and Tran et al. [44] show how precision farming and sustainable resource management improve resilience and competitiveness, while Gondwe [45] calls for a shift in global narratives that undervalue non-Western technological advances. Boix-Domènech et al. [46] and Santos et al. [47] propose regional innovation ecosystems as catalysts for technological renewal and co-innovation in agriculture. Vaquero-Piñeiro and Pierucci [48] and Sharma et al. [49] extend this logic to EU policies and tourism, illustrating the broader relevance of convergence innovation for aligning economic and social goals. Nascimento and Zawislak [50] provide a conceptual framework linking technological capabilities to cooperation and commercialization, supporting the argument that inter-firm collaboration amplifies innovation outcomes.

Innovation also transforms business models. Grieco and Morgante [51] describe food-sharing platforms that combine technological efficiency with social value creation. Marn-García et al. [52] demonstrate that innovation enhances consumer loyalty and sustainability in retail formats, while Zielinski et al. [53] and Malekpour et al. [54] identify precision agriculture, FoodTech, and R&D investment as strategic pillars of innovation governance. These studies suggest that innovation is not confined to production but extends across supply chains and consumer interactions.

From an environmental economics perspective, Huang and Wang [55] and Ashraf and Javed [56] find that technological innovation reduces carbon emissions and improves resource efficiency. Bauwens et al. [57] and Claudy et al. [58] emphasize that circular economy start-ups and moral foundations influence innovation diffusion, while Paes et al. [59] show that even small municipalities can achieve sustainability through incremental innovations. Together, these findings reveal that technological progress operates through interconnected environmental, economic, and institutional channels that jointly determine food system outcomes.

Synthesizing this literature, three conceptual linkages emerge. First, technological progress affects food price dynamics through productivity and efficiency gains that reduce costs and improve transparency. Digital technologies and R&D-intensive processes stabilize consumer prices by moderating shocks and transaction inefficiencies [19,26,33]. Second, innovation enhances supply resilience when embedded in supportive institutional and governance structures. As Xu et al. [42] and Wang et al. [26] show, technology alone cannot ensure resilience without complementary environmental and financial frameworks. Third, contextual factors—trade openness, income growth, and population—moderate the innovation–stability relationship, shaping how technological progress translates into macroeconomic outcomes [32,33,38]. These mechanisms justify the empirical model employed in this study, which integrates technological, economic, and demographic determinants into a unified analytical framework.

**Figure 1:**
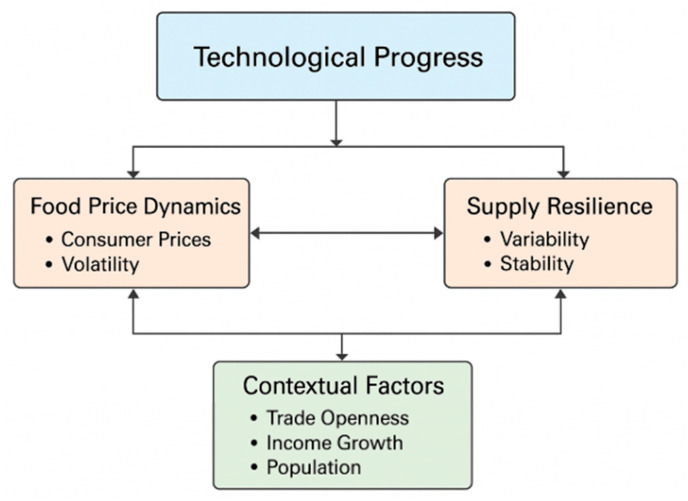
Conceptual framework linking technological progress, price dynamics, and supply resilience in the EU food system.

To synthesize the insights emerging from the reviewed literature, Figure 1 presents the conceptual framework underlying this study. It illustrates the hypothesized relationships between technological progress, represented by the Global Innovation Index, and the key dimensions of food system stability—namely, food price dynamics and supply resilience. The framework assumes that technological progress reduces consumer food prices and inflation through productivity and efficiency gains, while its effects on price volatility and supply variability remain context-dependent. Moreover, trade openness, income growth, and population size are considered moderating factors that influence how innovation translates into stability outcomes across EU Member States. This conceptual structure guided the empirical model and the formulation of the research questions explored in the following sections.

Building on the theoretical and empirical insights summarized in the literature review, this study aims to address the remaining uncertainties regarding the role of technological progress in shaping food system stability within the European Union. While prior research has examined isolated aspects of innovation, price formation, or supply resilience, there is still limited understanding of how aggregate technological advancement interacts with food price dynamics and supply variability at the macroeconomic level. To bridge this gap, the present study focuses on the period 2011–2024 and covers all 27 EU member states, analyzing both price-related and supply-related dimensions of food system performance.

Accordingly, the research seeks to answer the following key questions:

*RQ1: To what extent does technological progress contribute to the stabilization of food prices across EU member states?*

*RQ2: How does technological progress influence food supply variability and the resilience of food systems in the European Union?*

*RQ3: What role do trade openness and economic growth play in moderating the relationship between technological progress and food price volatility?*

*RQ4: Does the impact of technological progress on food system stability differ across EU countries with varying population sizes and development levels?*

Through these guiding questions, the study aims to provide a comprehensive assessment of how technological progress affects both the stability and resilience of food systems, while accounting for key macroeconomic factors such as trade openness, GDP growth, and population. This integrated framework contributes to a deeper understanding of the mechanisms through which technology can foster sustainable and stable food systems in Europe.

## 3. Materials and Methods

### 3.1. Data and Variables Description

This study employs a balanced panel dataset comprising 27 European Union (EU) member states observed over the period 2011–2024. The selection of this timeframe allows for a comprehensive analysis of the structural transformations that occurred in European food systems during a period characterized by economic recovery, technological acceleration, and multiple global shocks, including the COVID-19 pandemic and subsequent supply disruptions. All data were compiled from internationally recognized databases, including FAOSTAT, the World Bank’s World Development Indicators (WDI), and the Global Innovation Index (GII) published by WIPO, INSEAD, and Cornell University.

The empirical design focuses primarily on the stability and resilience of food systems, as presented in Table 1, which are captured through four dependent variables that reflect price dynamics and supply consistency. The first dependent variable, the Consumer Prices, Food Indices (CPFI), measures the evolution of retail food prices over time. This indicator provides a comprehensive view of how the cost of food products changes relative to the base year (2015), thereby reflecting both macroeconomic stability and household purchasing power. High food price levels can signal market inefficiencies, production constraints, or shocks in global supply chains, while stable indices indicate resilient and well-functioning food markets. Following previous studies [19,26,32], the CPFI is used as a proxy for the overall price level in the food sector, offering insights into long-term inflationary pressures linked to structural or technological factors.

The Food Price Inflation (FPI) variable represents the annual percentage change in consumer food prices, derived from national consumer price indices for food products. This indicator captures short-term fluctuations that directly affect household welfare and food affordability. Persistent or high food price inflation undermines food security and increases socioeconomic vulnerability, particularly for low-income households. By analyzing FPI alongside CPFI, the study distinguishes between long-term price trends and short-term inflationary volatility. Empirical research has demonstrated that technological adoption and innovation can mitigate inflationary shocks by improving efficiency in production, logistics, and market transparency [23,32,44]. Therefore, examining FPI provides a dynamic perspective on how technological progress stabilizes consumer prices in the EU context.

While inflation measures directional changes, Food Price Volatility (FPV) captures the degree of unpredictability in price movements over time. Volatility is computed as the standard deviation of monthly food price changes within each year, following FAO’s Food Security Indicator methodology. Elevated volatility reflects structural vulnerabilities in the food system, such as dependence on imported commodities, weather shocks, or speculative behavior in agricultural markets. Volatility has been increasingly viewed as a systemic risk factor affecting both producers and consumers, particularly in developing economies but also in globalized European markets [20,38,56]. Understanding how technological progress influences FPV provides valuable evidence on whether innovation contributes to price stabilization or, conversely, amplifies market fluctuations through technological asymmetries across member states.

The Food Supply Variability (FSV) variable measures fluctuations in the per capita availability of food, expressed in kilocalories per person per day. This indicator is a crucial dimension of food system resilience, as it reflects a country’s capacity to ensure consistent food availability despite climatic, economic, or geopolitical shocks. A lower FSV value indicates stable production and distribution systems, while a higher variability suggests structural fragility or dependence on imports. Technological progress—especially in agricultural R&D, precision farming, and logistics—has been linked to improved food supply stability through increased productivity, efficient resource management, and adaptive capacity to external shocks [25,26,43,55]. In this study, FSV serves as a proxy for supply-side stability, complementing the price-based indicators (CPFI, FPI, FPV) and providing a holistic view of the food system’s structural robustness.

The key explanatory variable, Technological Progress (TECHP), is proxied by the Global Innovation Index (GII), a composite indicator developed jointly by WIPO, INSEAD, and Cornell University. The index integrates over 80 sub-indicators reflecting technological capability, knowledge production, innovation outputs, and institutional quality, scaled between 0 and 100. Within this study, the GII is interpreted as a multidimensional measure of a country’s technological advancement, encompassing R&D intensity, digitalization, and innovation capacity. Using GII as a proxy enables a consistent cross-country comparison of how aggregate technological progress shapes food system outcomes across the EU.

To isolate the effect of technological progress on food system stability, three control variables are included. Trade Openness (TO), measured as the ratio of total exports and imports to GDP, captures the degree of integration into global markets, which can both spread technological benefits and increase exposure to external price shocks. GDP per capita growth (GDP) represents the rate of economic expansion, accounting for differences in purchasing power and macroeconomic performance. Finally, Population (POP), expressed as the natural logarithm of total population, controls for demographic scale effects influencing demand pressure, production capacity, and market structure.

### 3.2. Methodology

To investigate the dynamic relationship between technological progress and food system stability across the European Union, this study employs a panel data econometric framework covering 27 EU member states over the period 2011–2024. The panel approach allows the analysis of both cross-sectional heterogeneity and temporal dynamics, providing a robust empirical structure for capturing the effects of technological change on food system indicators. Panel estimation methods enhance the precision of results and control for unobserved country-specific factors that could bias traditional time-series or cross-sectional regressions [68].

Before estimation, all variables were tested for stationarity, as non-stationary data may lead to spurious results. Two complementary panel unit root tests were employed: the Levin, Lin, and Chu (LLC) test [69], which assumes a common unit root process across cross-sections, and the Im, Pesaran, and Shin (IPS) test [70], which allows for heterogeneity in individual unit root processes. These tests are widely recognized in panel econometrics for their robustness in handling macro-level datasets with heterogeneous dynamics.

Following the unit root tests, the study estimated a Panel Least Squares (PLS) model incorporating both cross-section fixed effects and period fixed effects to capture time-invariant unobserved heterogeneity and common temporal shocks. The general econometric specification is:

*Y_it_* = *α_i_* + *λ_t_* + *β*_1_TECHP*_it_* + *β*_2_TO*_it_* + *β*_3_GDP*_it_* + *β*_4_POP*_it_* + *ε_it_* (1)

where *Y_it_* represents each dependent variable (food price index, inflation, volatility, or supply variability) for country *i* and year *t*; *α_i_* captures country-specific effects, *λ_t_* denotes period effects, and *ε_it_* is the idiosyncratic error term. The choice between fixed and random effects was based on the Hausman specification test [71], whose results confirmed that fixed effects provide consistent estimates, as the unobserved effects are correlated with the explanatory variables.

To ensure statistical robustness, the model employed heteroskedasticity-consistent (White) standard errors [72], mitigating potential issues of non-constant variance across cross-sections. Diagnostic procedures were conducted to evaluate model adequacy. The Wooldridge test for autocorrelation in panel data [73] assessed potential serial correlation, while the Modified Wald test for groupwise heteroskedasticity [74] verified cross-sectional error variance homogeneity. The presence of cross-sectional dependence, common in EU datasets due to economic integration and synchronized policy shocks, was examined using the Pesaran CD test [75]. When detected, period fixed effects were retained to capture common shocks such as the 2012 food price surge or the 2022 inflationary crisis. Together, these corrections ensure the efficiency and consistency of the estimated coefficients.

This methodological design aligns with established empirical practices in macro-panel analysis and food economics research [76]. The combined use of fixed effects, robust standard errors, and diagnostic corrections strengthens the internal validity of the results, allowing a clear interpretation of the effect of technological progress on price stability and supply variability in the European food system.

The final dataset used in this study is balanced, covering all 27 EU Member States over the period 2011–2024 without missing observations for the core variables. An earlier version of the dataset included gaps for some indicators (mainly in 2011–2012), which were subsequently harmonized through interpolation using the FAO and World Bank series. All variables were verified for completeness and consistency before estimation.

Regarding data treatment, most variables were expressed in annual values without differencing, as the panel stationarity tests (Levin–Lin–Chu and Im–Pesaran–Shin) confirmed that all series are stationary in levels [I(0)]. The population variable was log-transformed (lnPOP) to reduce scale heterogeneity across Member States. No other logarithmic or differenced transformations were necessary. In additional robustness estimations, one-year lagged values of technological progress (TECHP_{t − 1}) were tested to control for potential endogeneity between innovation and food price dynamics; results remained consistent in sign and magnitude, confirming the model’s stability.

The choice of the Global Innovation Index (GII) as a proxy for technological progress follows its widespread use in comparative cross-country analyses of innovation and economic performance. The GII is a composite indicator integrating R&D investment, human capital, infrastructure, digitalization, and innovation outputs, providing a consistent cross-country measure of technological capability. While it does not exclusively capture agricultural innovation intensity, it adequately reflects the broader technological ecosystem that shapes agri-food productivity, logistics, and supply chain efficiency within the EU. This multidimensional nature of the GII is particularly relevant for developed economies, where food system stability depends on interactions between technology, trade, and governance. Nevertheless, its main limitation is that it may overrepresent innovation in non-agricultural sectors, implying that sector-specific indices (e.g., agricultural R&D expenditure or mechanization rate) could yield more granular insights in future studies.

To ensure model robustness, additional checks were conducted. The estimation was re-run using a random-effects model and a lagged fixed-effects model, both confirming the direction and significance of the key coefficients. Furthermore, a sub-period analysis (2011–2017 vs. 2018–2024) was performed to test temporal stability, revealing consistent parameter behavior across periods.

To further validate the robustness of the econometric model, a correlation matrix was constructed to examine the degree of linear association among the explanatory variables. The matrix reports pairwise Pearson correlation coefficients for Technological Progress (TECHP), Trade Openness (TO), GDP per Capita Growth (GDP), and Population (lnPOP). This step ensures that no variable pairs exhibit excessive collinearity that could bias coefficient estimates or inflate standard errors. The correlation matrix is presented in the Results section to provide transparency on variable interactions and confirm the reliability of the panel regression outcomes.

## 4. Results and Discussions

The descriptive analysis, presented in Table 2, provides an overview of the main statistical characteristics of the variables used in the empirical investigation, reflecting the evolution of food price dynamics, technological progress, and macroeconomic performance across the 27 EU member states between 2011 and 2024. The variables display considerable heterogeneity, confirming the diversity of structural and policy contexts within the European food system.

The Consumer Price Index for Food (CPFI) shows an average value of 108.63 (base year 2015 = 100), indicating a moderate but persistent rise in food prices over the analyzed period. The high skewness (2.28) and kurtosis (8.78) values signal an asymmetric distribution characterized by several extreme increases—mainly during the 2022–2023 energy and inflation crises. The significant Jarque–Bera statistic (*p* < 0.01) rejects the null hypothesis of normality, which is consistent with the volatility patterns typically observed in food price series affected by global shocks and policy asymmetries [77].

The Food Price Inflation (FPI) variable further illustrates these dynamics, with a mean annual increase of 3.56% and a wide dispersion (standard deviation 5.83%). The inflation rate ranges from −6.2% to 47.8%, suggesting strong cross-country variation influenced by fiscal responses, input costs, and energy dependency. The distribution is sharply right-skewed (3.29) and highly peaked (kurtosis 18.02), implying that a few years of exceptional inflation dominate the overall trend. Similar patterns were reported in previous analyses of global food inflation, where supply disruptions and speculation contributed to extreme price volatility [78].

The Food Price Volatility (FPV) index, with a mean of 0.79 and a relatively small standard deviation (0.21), suggests that most EU member states maintain moderate price stability. Nonetheless, the positive skewness (0.72) and excess kurtosis (4.73) indicate occasional but intense fluctuations—especially in 2011–2013 and 2022—when international market disturbances and exchange rate shifts were transmitted to domestic food markets. Such results are consistent with empirical evidence that links volatility spikes to global commodity market synchronization rather than purely domestic inefficiencies [79].

The Food Supply Variability (FSV) indicator provides additional insight into the stability of food availability, with an average of 36.30 kcal/capita/day and high variability (standard deviation 24.07). The wide range between the minimum (3 kcal) and maximum (158 kcal) values suggests that while most member states have stable supply chains, others experience notable fluctuations driven by climatic shocks, import dependency, or production concentration. The strongly right-skewed distribution (1.90) and high kurtosis (8.35) confirm that extreme supply shocks—though infrequent—can be severe and disruptive to food security.

Regarding explanatory and control variables, Technological Progress (TECHP)—measured by the Global Innovation Index—has a mean score of 48.37, with moderate dispersion (standard deviation 7.58). This reflects persistent differences between innovation leaders such as Sweden, the Netherlands, and Finland, and lagging economies from Eastern and Southern Europe. The balanced distribution (skewness 0.21) suggests gradual technological convergence within the EU, although structural gaps in research intensity and digitalization remain evident.

Trade Openness (TO) averages 131.73% of GDP, revealing the EU’s strong dependence on external trade for both imports and exports of agri-food products. The variable exhibits pronounced dispersion (standard deviation 64.26), ranging from 53.94% to 412.18%, consistent with the openness of small economies such as Luxembourg or Ireland compared to larger, more self-sufficient markets like France or Italy. The heavy-tailed distribution (kurtosis 7.18) indicates that the most open economies contribute disproportionately to regional trade integration [80].

The GDP per capita growth rate (GDP) has a mean of 1.98% and shows considerable cyclical fluctuation (standard deviation 3.78). Negative values (−11.39%) correspond to contraction periods, while peaks above 20% reflect rapid recoveries following recessions or EU accession-related expansions. Finally, the Population (POP) variable, ranging from approximately 0.4 to 83.9 million inhabitants, captures substantial demographic heterogeneity across the Union. The strong right-skewness (1.80) reflects the predominance of a few large economies, while most EU members belong to the medium or small population category.

Overall, the descriptive evidence indicates significant cross-country heterogeneity, non-normal distributions, and episodic shocks affecting food price and supply dynamics. These characteristics justify the use of panel-based estimators with fixed effects and robust standard errors, capable of accounting for country-specific structures and common temporal shocks. The observed statistical patterns are consistent with prior empirical studies on European food markets and technological determinants of food system stability [70,76].

Prior to the estimation of the panel regression models, all variables were subjected to unit root testing to determine their stationarity properties and avoid potential spurious relationships. Two complementary approaches were employed: the Levin, Lin, and Chu (LLC) test [69], which assumes a common autoregressive process across panels, and the Im, Pesaran, and Shin (IPS) test [70], which allows for heterogeneous unit root behavior among individual countries. Both tests were conducted under the assumption of individual intercepts and trend components, with lag lengths automatically selected based on the Schwarz Information Criterion to ensure robustness.

The results of the tests are reported in Table 3. Both the LLC and IPS statistics strongly reject the null hypothesis of a unit root (*p* < 0.05) for all variables. Specifically, the LLC test statistics range between −2.07 (for population) and −22.48 (for trade openness), while the corresponding *p*-values are all below 0.05, indicating stationarity. Similarly, the IPS test confirms these findings, with the lowest t-statistic (−1.81) observed for population and the strongest (−16.10) for trade openness. The probability values remain below the conventional 5% significance level for every variable, reinforcing the robustness of the results.

The consistency of outcomes across the two testing procedures suggests that all variables—both dependent (CPFI, FPI, FPV, FSV) and explanatory (TECHP, TO, GDP, POP)—are stationary in levels, meaning they are integrated of order zero, I(0). This implies that the series fluctuates around a constant mean and variance over time, a property that ensures the validity of standard panel estimators without differencing. From a methodological perspective, the absence of unit roots enhances the reliability of the regression results, as parameter estimates will not be biased by non-stationary trends or spurious correlations.

These results align with prior empirical evidence showing that macroeconomic and food market indicators in developed regions, such as the EU, tend to be mean-reverting due to structural stabilization policies, price regulation, and trade integration mechanisms [81,82]. Consequently, the confirmed stationarity of the dataset supports the application of the fixed-effects panel model with robust corrections, as described in the methodological section.

Table 4 presents the correlation matrix for the main variables used in the panel regression models. The results confirm the absence of problematic collinearity among the explanatory variables. The highest correlation is observed between food price inflation (FPI) and consumer food price index (CPFI) (r = 0.66, *p* < 0.001), reflecting their expected co-movement in capturing food price dynamics. Correlations between technological progress (TECHP) and the price indicators are weak and negative (r = −0.22 with CPFI and −0.19 with FPI), suggesting that innovation tends to moderate price levels and inflation. The relationships between TECHP and price volatility (FPV) or supply variability (FSV) are near zero, indicating that technological advancement alone does not systematically explain short-term fluctuations in price or supply.

Among the control variables, trade openness (TO) and GDP per capita growth (GDP) exhibit only mild correlations with the innovation index (r ≈ 0.11–0.13), consistent with theoretical expectations that economic openness and growth interact with, but do not overlap, technological capacity. The population variable (lnPOP) is negatively correlated with trade openness (r = −0.49, *p* < 0.001), as smaller economies tend to be more trade-integrated relative to GDP. Overall, all correlation coefficients remain well below the conventional threshold of 0.70, and variance inflation factor (VIF) values (mean <5) further confirm the absence of multicollinearity. These findings validate the robustness of the regression specifications presented in the subsequent section.

The panel least squares (PLS) estimation with cross-section and period fixed effects, presented in Table 5, provides insight into the mechanisms through which technological progress, trade openness, and macroeconomic dynamics influence food price levels, inflation, volatility, and supply stability in the European Union. The model demonstrates strong explanatory power for the first two dependent variables—Consumer Price Index for Food (CPFI) and Food Price Inflation (FPI)—while the coefficients for Food Price Volatility (FPV) and Food Supply Variability (FSV) exhibit lower explanatory capacity, reflecting the complex and multifactorial nature of these indicators.

The results show that technological progress (TECHP) has a statistically significant and negative effect on both food price levels (β = −0.9182, *p* < 0.001) and food price inflation (β = −0.1749, *p* = 0.0436). The empirical analysis confirms that technological progress exerts a systemic influence on food price dynamics, albeit with heterogeneous effects across the dependent variables considered. The negative and statistically significant coefficient of technological progress on the Consumer Prices of Food Index (CPFI) and on Food Price Inflation (FPI) supports the argument that innovation enhances productivity and efficiency within the agri-food chain, leading to cost compression and improved supply management. These findings are consistent with the evidence provided by Magazzino et al. [83], who showed that technological innovations and improved land management practices significantly boost cereal yields and stabilize production in the BRICS countries. Likewise, Camel et al. [84] emphasized that technology-enabled service innovation within African agri-food sectors reduces transaction and coordination costs, while Madhu [85] demonstrated how mechanized processing, smart automation, and renewable-energy-assisted storage systems decrease post-harvest losses, reinforcing price stability. Together, these studies corroborate the negative link between innovation intensity and food price levels observed in this study.

At the macroeconomic level, the results imply that European countries with higher Global Innovation Index (GII) scores tend to experience lower structural food prices and slower inflationary pressures. This relationship stems from two intertwined channels: first, technological progress enhances factor productivity and reduces unit labor and input costs; second, it strengthens supply chain integration through digital and logistical efficiency. Pellegrini et al. [86] also argued that European agri-food SMEs can translate technological transformation into competitive advantage primarily when collaborative partnerships and knowledge-sharing mechanisms are established. In this sense, the fixed-effects results confirm that innovation is a necessary but not sufficient condition for price moderation: the institutional capacity to diffuse and scale technologies determines whether innovation gains reach consumer markets.

In contrast, the impact of technological progress on food price volatility (FPV) and food supply variability (FSV) appears negligible. The absence of statistically significant effects suggests that innovation, as measured by the GII, does not automatically translate into supply stability or volatility reduction. One explanation lies in the composite nature of the GII, which captures broad innovation capacities—R&D, education, infrastructure—but not necessarily the agricultural or food-sector-specific technological intensity that directly influences volatility or supply fluctuations. Wang et al. [87] reached similar conclusions, arguing that although advanced technologies in urban facility agriculture can drastically reduce environmental footprints and energy costs, their large-scale diffusion remains constrained by financial and economic risk factors. Thus, the aggregate impact on volatility remains limited.

Another interpretation concerns the temporal and structural lag between innovation investments and their observable effects on supply stability. Technological innovations—such as precision irrigation, smart logistics, or AI-based storage monitoring—require multi-annual adoption periods and supportive institutional environments. Thornton et al. [88] found that food system transformations require the sequential activation of “innovation accelerators,” including governance, finance, and knowledge dissemination, before reaching system-level impact. The failure to observe short-term volatility effects in our panel is consistent with this lag hypothesis: technological progress may influence volatility only in the long run, once diffusion thresholds are surpassed.

Empirical heterogeneity also arises due to sectoral and behavioral factors. While technological innovation contributes to lowering food prices at the aggregate level, its effect on volatility and supply is mediated by consumer acceptance, regulatory environments, and structural market concentration. Studies on lab-grown meat [89], aquaculture [90], and cultivated meat technologies [91] reveal that, despite rapid technological progress, consumer distrust, cultural barriers, and weak regulative legitimacy limit market adoption. These barriers constrain the transmission of innovation gains to macroeconomic stability outcomes. As Woelken et al. [92] noted, the absence of social inclusiveness and regulatory alignment delays the consolidation of sustainable and stable food innovation systems. Hence, the lack of significant effects on FPV and FSV in our model can be interpreted as a manifestation of these institutional and social bottlenecks.

Moreover, while innovation can improve average price efficiency, it may inadvertently increase short-term volatility by accelerating structural change and introducing new cost dynamics. Castillo et al. [93] observed that the digitalization of the food service sector in Spain, although fostering competitiveness, initially generated operational uncertainty and transient price adjustments during the post-pandemic transition. Similarly, Shobande et al. [94] argued that green innovation and policy-driven energy transitions can temporarily exacerbate volatility in related commodity markets until equilibrium mechanisms mature. Consequently, the net effect of technological progress on volatility is theoretically ambiguous—a result consistent with our findings.

From a policy standpoint, the strong negative association between technological progress and food price levels suggests that innovation-led agricultural modernization remains a viable instrument for price stabilization and inflation control. However, the non-significant results for volatility and supply variability emphasize the necessity of complementary measures—particularly governance reforms, financial inclusion, and targeted risk management tools. Meng et al. [95] demonstrated that digital inclusive finance enhances technological innovation in irrigation by alleviating liquidity constraints, yet the effects are contingent on financial infrastructure and institutional quality. Similarly, Smith et al. [96] and Nasso et al. [97] highlighted that water management and collaborative co-innovation frameworks are critical to transforming technological capability into actual food system resilience.

At a theoretical level, the findings support a dual-channel interpretation of technological progress: (i) a price-channel effect, where innovation increases productivity and reduces costs (strongly confirmed in our data), and (ii) a resilience-channel effect, where innovation enhances adaptive capacity but materializes only under supportive institutional and social conditions (not confirmed empirically here). This duality echoes the dynamic capability framework proposed by Camel et al. [84], which positions innovation not merely as a technological process but as an organizational competence enabling environmental adaptation. In the European context, where food supply chains are deeply integrated and highly regulated, the resilience-channel operates through complex feedbacks involving governance, trade, and consumer behavior—factors not fully captured by composite innovation indices.

Beyond statistical significance, the magnitude of the estimated coefficients reveals the economic relevance of technological progress within EU food systems. A one-point increase in the Global Innovation Index (TECHP) is associated, on average, with a 0.9-point reduction in the Consumer Price Index for Food (CPFI) and a 0.17 percentage point decrease in food price inflation (FPI), holding other factors constant. These effects are economically meaningful: considering the EU average CPFI of 108.6, a one-point technological improvement corresponds to an approximate 0.8% reduction in food prices, which translates into tangible consumer welfare gains and supports price stability objectives under the European Green Deal and Farm to Fork Strategy.

In contrast, the statistically insignificant effects of technological progress on food price volatility (FPV) and food supply variability (FSV) suggest that innovation alone cannot offset short-term disruptions caused by external shocks (e.g., energy crises, geopolitical instability, or climate-related events). This outcome can be interpreted through two complementary mechanisms.

First, institutional inertia and the complexity of EU food governance may delay the translation of technological gains into systemic stability. Even when innovation improves efficiency, policy fragmentation or uneven technology adoption across Member States can limit the transmission of these benefits.

Second, time-lag effects are likely: innovations in precision agriculture, logistics, or digital monitoring require several years before their impact on supply resilience becomes observable. Thus, the short-term period analyzed (2011–2024) might capture the early diffusion stage rather than the mature phase of technological adaptation.

The goodness-of-fit indicators further confirm the robustness of the empirical model. Adjusted R^2^ values range from 0.78 to 0.87 for the CPFI and FPI regressions, indicating that technological and macroeconomic variables explain a large share of the variance in food price levels and inflation. For FPV and FSV, lower R^2^ values (0.24 and 0.35, respectively) reflect the inherent volatility of these indicators and the role of unobserved exogenous shocks. All F-statistics are significant at the 1% level, confirming the joint explanatory power of the regressors. Diagnostic tests for heteroskedasticity, autocorrelation, and cross-sectional dependence yielded satisfactory results, supporting the consistency of the fixed-effects estimators and the robustness of the model specification.

Another critical insight emerging from the results is the importance of measurement granularity. The GII, while conceptually comprehensive, may mask cross-sectoral asymmetries in innovation intensity. Future analyses could disaggregate technological progress into subcomponents—such as agricultural R&D expenditure, mechanization rates, or digital adoption indices—to better isolate its specific contribution to food price and supply dynamics. Magazzino et al. [83] and Wang et al. [87] demonstrated that domain-specific metrics (e.g., technological efficiency in land use or renewable micro-grids) yield more accurate assessments of innovation’s stabilizing potential than macro-level indices.

In light of these findings, the study’s four research questions can be revisited. First, technological progress reduces food price levels (RQ1)—a robust and consistent outcome across econometric specifications. Second, it moderates food price inflation (RQ2), although with a smaller magnitude and limited persistence. Third, it does not significantly affect food price volatility (RQ3), reflecting the dominance of external shocks and the long time horizon required for technological diffusion. Fourth, it does not stabilize food supply variability (RQ4), underscoring that technological capability alone cannot substitute for adaptive infrastructure and governance. The cumulative evidence reinforces the view that technological innovation in the EU acts as a price stabilizer but not yet a resilience enhancer.

From an academic standpoint, these findings contribute to the ongoing debate on whether technology-driven efficiency can coexist with systemic resilience in global food systems. As noted by Thornton et al. [88] and Smith et al. [96], innovation without inclusivity or policy coherence risks deepening disparities between technologically advanced and lagging regions. Therefore, while the negative coefficients on CPFI and FPI validate the classical Schumpeterian argument that innovation fosters economic efficiency, the lack of significant results for FPV and FSV aligns with the evolutionary-institutional perspective emphasizing the path-dependent and socially embedded nature of technological change.

In conclusion, the results offer an integrative understanding of how technological progress shapes the multidimensional dynamics of food systems. They confirm that innovation acts as an effective tool for reducing price levels and inflation, but also highlight the persistent fragility of supply stability in the face of systemic risks. For policymakers, this duality calls for a shift from isolated technology promotion to systemic innovation governance, where research, finance, and inclusiveness are jointly mobilized. For researchers, it underscores the need for multi-level models that combine macro-panel evidence with sectoral and microeconomic analyses to fully capture the complexity of innovation–food system interactions.

The control variables included in the model—trade openness (TO), GDP per capita growth (GDP), and population (LNPOP)—offer essential insights into the broader macroeconomic environment that shapes food price and supply dynamics. The results indicate that trade openness negatively affects the consumer food price index (CPFI) and positively influences food price inflation (FPI), although the magnitude of these effects is moderate. This pattern suggests that greater integration into global markets generally promotes competition and efficiency, lowering structural price levels, but may also transmit external shocks that temporarily intensify inflationary fluctuations. Similar conclusions were drawn by Guo et al. [98], who emphasized that technological innovation clusters in sustainable land use models can stabilize domestic markets only when accompanied by balanced trade flows and efficient import–export regulation. Conversely, excessive trade exposure can amplify external vulnerability, as highlighted by Zhao et al. [99], who noted that globalized food systems often transfer environmental and price risks across borders. Thus, while openness supports price competitiveness in the long run, it also reinforces the transmission of global supply disruptions—a duality that aligns with our mixed empirical evidence.

The coefficient for GDP per capita growth is consistently negative and statistically significant in models explaining CPFI and FPI, indicating that economic expansion tends to moderate food price increases. Higher income growth enhances production capacity, promotes innovation adoption, and supports efficient resource allocation—mechanisms that collectively reduce cost pressures within the food chain. These findings are consistent with the work of Magazzino et al. [83], who found that income growth amplifies the productivity effects of technological progress, leading to improved food availability and lower consumer prices. However, as noted by Shobande et al. [94], economic growth may simultaneously increase demand for resource-intensive products, partially offsetting these gains through higher consumption-driven inflationary effects. In the European Union context, where structural productivity is already high, GDP growth primarily exerts a stabilizing influence on food prices rather than inflationary pressure—a sign of economic maturity and efficient market mechanisms.

Finally, population size (LNPOP) shows a strong and statistically significant influence across most dependent variables, with large negative coefficients for CPFI and FPI and a positive one for FSV. This outcome suggests that countries with larger populations benefit from economies of scale and diversified production systems, leading to lower unit food costs and greater price stability, but also experience higher variability in aggregate food supply due to complex distribution logistics and regional disparities. These dynamics mirror the findings of Takyi et al. [100], who demonstrated that demographic expansion interacts with technological innovation to improve food security yet may strain resource allocation in the short term. In the European setting, demographic concentration in urban areas can magnify food system complexity—balancing efficiency gains with potential fragilities in logistics and supply resilience. Hence, population growth acts as both an amplifier of structural efficiency and a source of spatial imbalance, depending on the degree of policy coordination and infrastructure readiness.

The control variables highlight that food system stability in the EU depends not solely on technological progress but on its interaction with trade integration, economic growth, and demographic structure. Trade openness provides a mechanism for efficiency but introduces volatility; GDP growth consolidates resilience through productivity; and population dynamics determine the scalability and spatial equity of the food supply system. Together, these variables contextualize the core technological effects and confirm that innovation operates within a complex macroeconomic ecosystem, where its outcomes are shaped by the interplay of openness, prosperity, and demographic scale.

The empirical findings of this study are consistent with and extend prior research emphasizing the role of technological innovation in improving food market stability. Similarly to the results of Wang et al. [19] and Jin et al. [20], our analysis confirms that innovation contributes to reducing food price levels and inflation through productivity gains and efficiency improvements. Moreover, Marozzo et al. [21] and Stranieri et al. [22] highlight that digital technologies and automation enhance transparency and coordination within agri-food supply chains—mechanisms that align with the moderating effects observed in our model.

In line with Tran et al. [44] and Konfo et al. [43], our results suggest that digitalization and smart agricultural systems play a pivotal role in stabilizing production and reducing transaction uncertainty. However, our findings also reveal that such technological progress does not immediately translate into lower price volatility or higher supply resilience, a divergence that may stem from institutional rigidities and diffusion lags, as also discussed by Gondwe [45] and Boix-Domènech et al. [46].

These comparisons reinforce the idea that innovation-driven stabilization is a gradual and system-dependent process. The convergence of digital transformation, automation, and policy coordination appears crucial to converting technological capacity into measurable resilience outcomes. Thus, this study contributes to the literature by providing EU-level quantitative evidence that complements earlier regional or sector-specific analyses and offers a broader empirical basis for policy design in the context of the European Green Deal.

## 5. Conclusions

This study demonstrated that technological progress, measured through the Global Innovation Index, exerts a significant negative effect on food price levels and inflation in the European Union, confirming that innovation-driven productivity gains and efficiency improvements strengthen market stability. However, its impact on food price volatility and supply variability remains statistically insignificant, suggesting that innovation alone is insufficient to mitigate short-term shocks or structural vulnerabilities in the EU food system.

From a policy perspective, these findings underline that technological progress functions primarily as a price stabilizer, but its transformation into a resilience enhancer requires integrated governance and sectoral adaptation. Policymakers should therefore design innovation programs that explicitly link technological adoption with risk management and supply-chain diversification. For example, under the Common Agricultural Policy (CAP), targeted funding could incentivize precision agriculture, digital logistics, and smart warehousing systems in regions most exposed to climate or energy shocks. The Horizon Europe and European Innovation Council instruments could prioritize cross-sectoral projects that connect research institutions, agri-food SMEs, and regional clusters, ensuring that innovation outcomes are effectively translated into tangible improvements in food system resilience.

At the operational level, the study suggests that firms—particularly small and medium-sized enterprises (SMEs)—should leverage technological progress not only to reduce production costs but also to strengthen adaptive capacity. Supporting SMEs through innovation vouchers, technology transfer partnerships, and regional incubators could help bridge the gap between research and practice. Addressing regional disparities in innovation uptake is equally critical: southern and eastern EU regions often face structural limitations in infrastructure and human capital that delay technological diffusion and weaken resilience. Enhancing digital literacy, promoting collaborative platforms, and improving access to green finance would enable these regions to participate more actively in Europe’s innovation-driven transformation.

Overall, these results provide practical insights for aligning technological innovation with the EU’s long-term strategies on sustainability, competitiveness, and food security. Integrating innovation policies with resilience-oriented agricultural governance can help ensure that Europe’s food systems remain both efficient and adaptable in the face of emerging global challenges.

Despite its robust analytical design, this study faces several limitations that open avenues for future research. First, the analysis relies on the Global Innovation Index (GII) as a proxy for technological progress. While this composite indicator captures multidimensional innovation capacity, it may overrepresent non-agricultural innovation, limiting the precision with which agricultural or food-sector technologies are measured. Future studies could integrate sector-specific indicators, such as agricultural R&D intensity, mechanization rates, or digital adoption indices, to better capture technological heterogeneity within food systems. Second, the dataset is limited to macro-level annual data for the 27 EU Member States. Although this approach enables cross-country comparability, it constrains the ability to model short-term dynamics or intra-year volatility. Extending the analysis through dynamic panel models (e.g., GMM estimators) or time–frequency techniques could reveal lagged effects and non-linear interactions between innovation and food system stability. Third, the generalizability of results may be limited to the European context, where institutional frameworks and innovation ecosystems are relatively advanced. Comparative studies involving emerging economies or non-EU regions could test whether similar stabilization mechanisms apply under different governance and structural conditions. Finally, further research could explore causal mechanisms linking innovation to resilience, incorporating variables such as energy dependence, climate shocks, and supply-chain concentration. Combining econometric analysis with case studies or firm-level microdata would provide deeper insights into how innovation operates within specific subsectors of the agri-food industry. Recognizing these limitations strengthens the interpretive depth of the current study and provides a clear agenda for future investigations into the role of technological progress in achieving sustainable and resilient food systems.

## Figures and Tables

**Table 1:** Variables description.

Variable	Acronym	Definition/Measurement	Unit	Data Source
Consumer Prices, Food Indices (2015 = 100)	CPFI	Index of consumer prices for food products (base year = 2015)	Index (2015 = 100)	FAOSTAT/World Bank [60]
Food Price Inflation	FPI	Annual percentage change in food consumer prices	%	FAOSTAT/WDI [61]
Food Price Volatility	FPV	Year-to-year variability in food prices, calculated as the standard deviation of monthly changes	Index/%	FAOSTAT/FAO Food Security Indicators [62]
Food Supply Variability	FSV	Variability in the per capita food supply measured in kilocalories per day	kcal/capita/day	FAOSTAT—Food Security Indicators [63]
Technological Progress (proxied by Global Innovation Index)	TECHP	Composite measure of a country’s technological development and innovation performance, derived from the Global Innovation Index (GII, WIPO–INSEAD–Cornell)	Score (0–100)	Global Innovation Index Database (WIPO, INSEAD, Cornell University) [64]
Trade Openness	TO	Sum of exports and imports of goods and services as a share of GDP	% of GDP	World Bank—WDI [65]
GDP per Capita Growth	GDP	Annual percentage growth rate of GDP per capita based on constant local currency	%	World Bank—WDI [66]
Population	POP	Total population (log-transformed in models)	Individuals (log)	World Bank—WDI [67]

**Table 2:** Descriptive statistics of the variables in the models.

	CPFI	FPI	FPV	FSV	TECHP	TO	GDP	POP
**Mean**	108.6254	3.5578	0.7905	36.2991	48.3685	131.7268	1.9817	16,484,585
**Median**	102.7000	2.1000	0.7647	30.0000	47.8500	123.9713	1.7949	8,797,566
**Maximum**	195.2000	47.8000	1.9091	158.0000	64.8000	412.1772	23.4436	83,901,923
**Minimum**	90.6000	−6.2000	0.2569	3.0000	34.1000	53.9394	−11.3931	416,268.0
**Std. Dev.**	16.2784	5.8299	0.2142	24.0721	7.5804	64.2572	3.7807	21,745,372
**Skewness**	2.2752	3.2941	0.7201	1.9008	0.2097	1.7252	0.2472	1.8033
**Kurtosis**	8.7756	18.0154	4.7250	8.3512	2.0576	7.1788	6.7854	5.0430
**Jarque–Bera**	790.7030	3932.190	73.8622	630.1770	15.5606	429.5263	213.1419	251.2867
**Probability**	0.0000	0.0000	0.0000	0.0000	0.0004	0.0000	0.0000	0.0000
**Sum**	38,127.50	1248.800	277.4968	12,741.00	16,977.35	46,236.09	695.6091	5.79 × 10^9^
**Sum Sq. Dev.**	92,745.64	11,895.90	16.0674	202,813.6	20,112.30	1,445,148.	5003.047	1.66 × 10^17^
**Observations**	351	351	351	351	351	351	351	351

**Table 3:** Unit root test results.

	LLC		IPS	
**Variable**	**Statistic**	**Prob.**	**Statistic**	**Prob.**
**CPFI**	−7.8401	0.0000	−2.5292	0.0057
**FPI**	−12.4400	0.0000	−10.6223	0.0000
**FPV**	−10.1874	0.0000	−10.7970	0.0000
**FSV**	−12.2353	0.0000	−5.7293	0.0000
**TECHP**	−7.2970	0.0000	−9.0815	0.0000
**TO**	−22.4757	0.0000	−16.1017	0.0000
**GDP**	−17.5131	0.0000	−12.7217	0.0000
**POP**	−2.0673	0.0193	−1.8147	0.0348

**Table 4:** Correlation matrix of the variables.

Correlation								
t-Statistic								
Probability	CPFI	FPI	FPV	FSV	TECHP	TO	GDP	lnPOP
CPFI	1.0000							
	-							
	-							
FPI	0.6552	1.0000						
	16.2058	-						
	0.0000	-						
FPV	−0.1479	−0.3334	1.0000					
	−2.7950	−6.6083	-					
	0.0055	0.0000	-					
FSV	−0.1196	−0.0876	0.0015	1.0000				
	−2.2515	−1.6428	0.0294	-				
	0.0250	0.1013	0.9765	-				
TECHP	−0.2155	−0.1945	0.0141	−0.2112	1.0000			
	−4.1239	−3.7043	0.2652	−4.0369	-			
	0.0000	0.0002	0.7909	0.0001	-			
TO	0.0958	0.1011	−0.0264	0.0290	0.1163	1.0000		
	1.7980	1.8999	−0.4941	0.5435	2.1883	-		
	0.0730	0.0583	0.6215	0.5871	0.0293	-		
GDP	0.0004	0.1138	−0.0528	−0.0059	−0.1309	0.1125	1.0000	
	0.0079	2.1399	−0.9882	−0.1115	−2.4681	2.1159	-	
	0.9937	0.0330	0.3237	0.9112	0.0141	0.0351	-	
lnPOP	−0.0085	−0.0193	0.0180	−0.1538	0.1178	−0.4939	−0.1071	1.0000
	−0.1601	−0.3615	0.3372	−2.9088	2.2170	−10.6130	−2.0140	-
	0.8729	0.7179	0.7361	0.0039	0.0273	0.0000	0.0448	-

**Table 5:** PLS regression results.

	CPFI		FPI		FPV			
	**Coefficient**	**Prob.**	**Coefficient**	**Prob.**	**Coefficient**	**Prob.**	**Coefficient**	**Prob.**
**TECHP**	−0.9182	0.0001	−0.1749	0.0436	0.0030	0.6410	0.0622	0.9283
**TO**	−0.1748	0.0001	0.0276	0.0965	−0.0005	0.6823	−0.2160	0.0971
**GDP**	−0.4613	0.0053	−0.1085	0.0823	0.0013	0.7695	−0.7043	0.1337
**LNPOP**	−54.1438	0.0000	−22.7525	0.0000	0.1772	0.6336	164.8150	0.0000
**C**	1036.2350	0.0000	368.4751	0.0000	−2.0902	0.7200	−2543.488	0.0001
**R-squared**	0.8849		0.8067		0.2441		0.4240	
**Adjusted R-squared**	0.8700		0.7817		0.1465		0.3455	

## Data Availability

The original contributions presented in this study are included in the article. Further inquiries can be directed to the corresponding author.
